# Identifying responders to gabapentin for the treatment of alcohol use disorder: an exploratory machine learning approach

**DOI:** 10.1093/alcalc/agaf010

**Published:** 2025-03-26

**Authors:** Lara A Ray, Erica N Grodin, Wave-Ananda Baskerville, Suzanna Donato, Alondra Cruz, Amanda K Montoya

**Affiliations:** Department of Psychology, University of California, Los Angeles, 1285 Franz Hall, Los Angeles, CA 90095, United States; Brain Research Institute, University of California, Los Angeles, 695 Charles E Young Drive S, Los Angeles, CA 90095, United States; Department of Psychiatry and Biobehavioral Sciences, University of California, Los Angeles, 760 Westwood Plaza, Los Angeles, CA 90095, United States; Department of Psychology, University of California, Los Angeles, 1285 Franz Hall, Los Angeles, CA 90095, United States; Brain Research Institute, University of California, Los Angeles, 695 Charles E Young Drive S, Los Angeles, CA 90095, United States; Department of Psychiatry and Biobehavioral Sciences, University of California, Los Angeles, 760 Westwood Plaza, Los Angeles, CA 90095, United States; Department of Psychology, University of California, Los Angeles, 1285 Franz Hall, Los Angeles, CA 90095, United States; Department of Psychology, University of California, Los Angeles, 1285 Franz Hall, Los Angeles, CA 90095, United States; Department of Psychology, University of California, Los Angeles, 1285 Franz Hall, Los Angeles, CA 90095, United States; Department of Psychology, University of California, Los Angeles, 1285 Franz Hall, Los Angeles, CA 90095, United States

## Abstract

**Background:**

Gabapentin, an anticonvulsant medication, has been proposed as a treatment for alcohol use disorder (AUD). A multisite study tested gabapentin enacarbil extended-release (GE-XR; 600 mg/twice a day), a prodrug formulation, combined with a computerized behavioral intervention, for AUD. In this multisite trial, the gabapentin GE-XR group did not differ significantly from placebo on the primary outcome of percent of subjects with no heavy drinking days. Despite the null findings, there is considerable interest in using machine learning methods to identify responders to GE-XR. The present study applies interaction tree machine learning methods to identify positive and iatrogenic (i.e. individuals who responded better to placebo than to GE-XR) treatment responders in the trial.

**Methods:**

Baseline characteristics taken from the multisite trial were examined as potential moderators of treatment response using qualitative interaction trees (QUINT; *N* = 338; 223 M/115F). QUINT models are an exploratory decision tree approach that iteratively splits the data into leaves based on predictor variables to maximize a specific criterion.

**Results:**

Analyses identified key factors that are associated with the efficacy (or iatrogenic effects) of GE-XR for AUD. Such factors are baseline drinking levels, motivation for change, confidence in their ability to reach drinking goals (i.e. self-efficacy), cognitive impulsivity, and baseline anxiety levels.

**Conclusion:**

Baseline drinking levels and anxiety levels may be associated with the protracted withdrawal syndrome, previously implicated in the clinical response to gabapentin. However, these analyses underscore motivation for change and self-efficacy as predictors of clinical response to GE-XR, suggesting these established constructs should receive further attention in gabapentin research and clinical practice. Multiple studies using different machine learning methods are valuable as these novel analytic tools are applied to medication development for AUD.

## Introduction

Gabapentin is an anticonvulsant medication approved by the United States Food and Drug Administration (FDA) for the treatment of partial epileptic seizures and for postherpetic neuralgia. Gabapentin’s mechanism of action is as a calcium channel–aminobutyric acid modulator and has been available as a generic medication since 2004. Unlike medications for alcohol use disorder (AUD) with anticraving properties, such as naltrexone (Hendershot *et al*. 2017) and topiramate (Guglielmo *et al*. 2015), gabapentin may exert its effects through alleviation of alcohol withdrawal. In fact, a trial comparing pregabalin to naltrexone found that pregabalin resulted in greater improvement in symptoms such as anxiety, hostility, and psychoticism (Martinotti *et al*. 2010).

The application of gabapentin for the treatment of AUD spans over two decades (Mason *et al*. 2018). For instance, studies have highlighted the efficacy of gabapentin for the treatment of alcohol withdrawal symptoms (Mariani *et al*. 2006; Myrick *et al*. 2009). Additional trials found a benefit of combining gabapentin with naltrexone (Anton *et al*. 2011) and with flumazenil (Anton *et al*. 2009; Schacht *et al*. 2011). These studies consistently emphasized the role of alcohol withdrawal symptoms as a treatment target for gabapentin and as a predictor of treatment response. Namely, individuals reporting alcohol withdrawal symptoms appear more likely to benefit from gabapentin.

Three pivotal randomized clinical trials tested the overall efficacy of gabapentin for the treatment of AUD. Mason *et al*. (2014) tested oral gabapentin at 900 or 1800 mg/day, versus placebo, combined with manualized counseling, and found that the 1800 mg dosage was effective in promoting abstinence, reducing heavy drinking, improving sleep, and decreasing alcohol craving (Mason *et al*. 2014). Anton *et al*. (2020) tested gabapentin (up to 1200/mg/day), versus placebo, combined with medical management and found that the clinical benefits of gabapentin were apparent in the high-alcohol withdrawal group only (Anton *et al*. 2020). A multisite study by the National Institute on Alcohol Abuse and Alcoholism (NIAAA) Clinical Investigators Group (NCIG) tested gabapentin enacarbil extended-release (GE-XR; 600 mg/twice a day), a prodrug formulation, combined with a computerized behavioral intervention (Take Control, a computerized behavioral platform; Devine *et al*. 2016), for AUD (Falk *et al*. 2019). This large-scale trial found that the gabapentin GE-XR group did not differ significantly from placebo on the primary outcome measure of percent of subjects with no heavy drinking days. Falk *et al*. (2019) examined 26 potential moderators and found that treatment drinking goal and attentional impulsivity moderated the effect of treatment. Specifically, GE-XR was more efficacious for individuals with a nonpermanent abstinence treatment goal and with low impulsivity scores, compared to placebo. The authors posit that low gabapentin exposure in the GE-XR formulation may account for the null findings, as the lower exposure was supported by pharmacokinetics analysis (Falk *et al*. 2019). Notably, the participants in the NCIG trial had low levels of alcohol withdrawal at baseline, which may have impacted the null findings.

Furthermore, there is a favorable perception of gabapentinoids by prescribers, who often use them for off-label indications (Kim *et al*. 2018; Huang *et al*. 2023). Thus, while the large-scale multisite trial of GE-XR did not produce the efficacy findings that would support its indication for AUD treatment, there is considerable interest in identifying responders to GE-XR. To that end, Laska *et al*. (2020) conducted a likely responder reanalysis of the Falk *et al*. (2019) clinical trial. In their study, the primary outcome used to identify likely responders was reduction from the baseline of the number of heavy drinking days. Analyses using a random forest model found that likely responders to GE XR had a higher number of heavy drinking days at baseline, lower levels of anxiety and depression, and higher cognitive and motor impulsivity (Laska *et al*. 2020).

This application of machine learning methods to a pivotal clinical trial of gabapentin for AUD is well reasoned yet has notable limitations. First, the analytic approach used by Laska *et al*. (2020) does not directly identify the specific variables that contribute to differentiating participants as likely or unlikely responders and *post hoc* analyses are required to identify these variables. Because of the *post hoc* nature, this approach doesn’t account for collinearity among potential moderators and so cannot uniquely distinguish the role of correlated moderators. Second, Laska and colleagues used a 14-day reduction in heavy drinking days as a cut-off for identifying treatment responders. While this cut-off is clinically based, it is arbitrary to some degree, and there may be ways to identify participants as “treatment responders” who saw reductions smaller than 14 days. About 10% of participants had fewer than 14 heavy drinking days (HDD) at baseline, making it impossible to identify them as treatment responders. Notably, if the goal is to identify treatment responders and nonresponders, this is a patient-focused goal rather than a variable-focused goal. In other words, the goal of the study is to categorize participants as treatment responders or nonresponders, rather than focus on which variables are the strongest predictors of treatment response. If participants are unable to be classified into one category based on outcome variable definitions, the goal of identifying treatment responders and iatrogenic responders is unable to be successful. Iatrogenic responders are defined as individuals who have better clinical outcomes on a placebo, compared to the active medication. Both the methods applied by Falk *et al*. (2019) and Laska *et al*. (2020) focus on identifying which variables moderate the treatment effect. The QUINT (Qualitative Interaction Tree) approach, which we apply here, focuses on identifying groups of individuals with similar treatment effects, subsetting participants into groups of treatment responders, nonresponders, and iatrogenic responders.

The QUINT approach provides five concrete advantages over prior approaches applied to this data. First, the QUINT approach allows for complex interactions and is a nonparametric model allowing also for nonlinear patterns of change in the treatment response along the range of the moderators. By examining complex interactions, we can identify combinations of moderators that might work together to lead to treatment response type; this is a strong advantage over the approach by Falk *et al*. (2019), which investigated individual moderators, but a property shared with Laska *et al*. (2020). Second, QUINT differentiates iatrogenic responders (i.e. people who are expected to have a significantly worse response to treatment than placebo) from nonresponders (i.e. people who are expected to have no different response to treatment than placebo). This property is a strong advantage over Falk *et al*. (2019), which did not classify responders at all and over Laska *et al*. (2020), which only differentiated treatment responders from others. Third, the method directly identifies specific moderators that can be used to differentiate types of responders, and these moderators are all evaluated simultaneously rather than individually, meaning that the method accounts for collinearity among moderators. This variable identification process is very important because it would allow clinicians to determine which measures to collect with clients in order to determine treatment action, rather than performing a broad battery that could be very time intensive. The approach proposed by Laska *et al*. (2020) requires inclusion of all variables in the estimation of the propensity scores, which could be prohibitively costly in healthcare settings. Fourth, there is no need to select a specific cut-off to define treatment responders; rather, the algorithm identifies which groups can be classified as treatment responders based on the data. Finally, the QUINT method is a participant-focused analysis, meaning that it is searching for the optimal partitioning of participants rather than a variable-focused analysis, which suggests that this method should have stronger predictive accuracy than variable-focused methods (Lipkovich *et al*. 2017). In general, we believe that the QUINT approach is particularly appropriate for detecting types of treatment responders. Furthermore, since the field is in its early stages of applying machine learning (ML) methods to clinical trials, we argue that having multiple ML methods applied to pivotal trials is crucial to leveraging these analytic tools. Therefore, the present study examines the clinical trial data from Falk *et al*. (2019) and applies QUINT methods to identify positive and iatrogenic treatment responders in the trial using baseline clinical and demographic measures.

## Materials and method

### Study overview

Data for this secondary analysis were drawn from a 26-week, multisite Phase 2 double-blind, placebo-controlled, parallel group trial of gabapentin enacarbil extended-release (GE-SR) (NCT0225236). This data set was publicly available to qualified investigators and was obtained by the first author (L.A.R.) through a material transfer agreement with the NCIG/NIAAA. Participants were randomly assigned to GE-XR or matched placebo on a 1:1 ratio. Randomization was stratified by clinical site using a permuted block randomization procedure. GE-XR was supplied in 600 mg tablets that contained an equivalent of 313 mg of gabapentin. GE-XR dosage was titrated as follows: a starting dose of one tablet (600 mg) once a day on Days 1–3 and the target dose of two tablets (600 mg twice a day) on Days 4–7 and Weeks 2–25, followed by a taper to one tablet (600 mg) once a day during Week 26.

### Study population

Randomized study participants were 338 individuals (66% males/34% females) with past-year moderate to severe AUD according to the DSM-5 (American Psychiatric Association, 2013). Study inclusion criteria were: (i) at least 21 years of age; (ii) reported drinking ≥21 standard drinks per week on average for women and ≥28 standard drinks per week on average for men; (iii) had ≥1 heavy drinking day per week during the 28-day period before consent; and (iv) ≥3 consecutive days of abstinence prior to randomization. Exclusion criteria included meeting DSM-5 criteria for a substance use disorder or psychiatric disorder; contraindicated medical conditions; and use of psychiatric medications (see Falk *et al*. (2019) for complete inclusion and exclusion criteria and participant demographics).

### Measures

#### Primary outcomes

The current analysis used the primary drinking endpoints from the original randomized controlled trial (RCT). The maintenance phase of the trial was defined as Weeks 2–25, postmedication titration. The primary efficacy outcome for the trial was a binary outcome measure of zero heavy drinking days during the last 4 weeks (Weeks 22–25) of the maintenance phase of the trial. Heavy drinking days were defined as more than or equal to four standard drinks for females and more than or equal to five standard drinks per day for males. To leverage a broader characterization of change in drinking behaviors, the present study used both raw change in heavy drinking days and proportion change in heavy drinking days during the maintenance phase of the trial as the primary outcome measures [baseline – maintenance)/baseline]. All drinking measures were captured through the Timeline Followback (TLFB; Sobell *et al*. 1992).

#### Demographics

Baseline demographic variables were assessed during the initial screening visit. Variables included in the present analysis were sex (M/F), education (years), and annual household income. Race was not included in the model as recommended to mitigate health disparities associated with the use of data-driven methods (Paulus and Kent 2020).

#### Alcohol use variables

Baseline alcohol use patterns were captured using the TLFB and included the number of heavy drinking days in the 28-day period prior to screening, the number of drinks per drinking day in the past 28-day period, the number of standard drinks per week, the number of standard drinks per drinking day, and percent days abstinent during the 30 days prior to screening. The following variables assessed alcohol use severity and history: years of regular drinking (current age – age of onset); AUD symptom count via the DSM-5; alcohol craving measured through the Alcohol Craving Questionnaire (Singleton *et al*. 1994); negative drinking-related consequences score assessed via the ImBIBE, which is an adaptation of the Drinkers Inventory of Consequences (Miller 1995; Werner *et al*. 2008); alcohol withdrawal severity assessed using the Clinical Institute Withdrawal Assessment for Alcohol (Sullivan *et al*. 1989); endorsement of two or more alcohol withdrawal symptoms was assessed via the DSM-5; and the Thoughts About Abstinence Scale (Marlatt *et al*. 1988) assessed drinking goal, motivation to meet the selected goal, and confidence in reaching the desired goal.

#### Behavioral measures

The following behavioral measures were collected at baseline: the Barratt Impulsiveness Scale (Patton *et al*. 1995) to measure second-order factors of impulsiveness such as attention, motor, and nonplanning impulsiveness; the Beck Anxiety Inventory (Beck *et al*. 1993); the Beck Depression Inventory-II (Beck *et al*. 1996); the Profile of Mood States (McNair 1992); and the Pittsburgh Sleep Quality Index (Buysse *et al*. 1989), with higher scores indicating poorer sleep quality.

#### Cigarette smoking

Smoking status was indexed by the number of cigarettes per week at baseline, such that more than or equal to one cigarette per week indicated an active smoking status.

### Statistical analysis

QUINT were used to predict two primary outcomes: raw change in percent heavy drinking days (to more closely align with Laska *et al*. 2020) and proportion change in percent heavy drinking days [(baseline – maintenance)/baseline]. QUINT models are an exploratory decision tree approach that iteratively splits the data into leaves based on predictor variables in a way that maximizes a specific criterion (Dusseldorp and Van Mechelen 2014; Dusseldorp *et al*. 2016). What differentiates QUINT from traditional decision trees is that decision trees optimize similarity on the outcome variable, creating leaves of participants that have similar scores on the outcome. QUINT, on the other hand, aims to create leaves of participants that have similar estimated treatment effects. This is achieved by balancing two components: the mean difference between groups within a leaf and the number of participants in each leaf. The data are iteratively partitioned until the splitting criteria cannot be improved. The splitting criteria’s standard error is estimated with a bias-corrected bootstrap using 200 bootstrap samples. The models are pruned to only allow for as many leaves as significantly improve the splitting criteria. Finally, each leaf is categorized into one of three categories: treatment responders (represented in green), iatrogenic responders (represented in red), and nonresponders (represented in taupe). We fit the models with a maximum of eight leaves for better predictive performance. All demographics (except race), alcohol use, behavior, smoking, and compliance measures described above were used as predictors in the models, aligning with Laska *et al*. (2020).

In our analysis, missing data were handled differently for predictors and outcomes. For the predictors, when individual items were missing or marked “Refuse to Respond,” an imputation model was used to fill in these responses based on responses to all other predictors^1^. Two exceptions were items on the ImBIBE survey involving parenting (25% missing) and marriage/love relationships (10% missing). Because of the high level of missingness on these items, driven by “Not Applicable” responses, these items were not used in scoring the ImBIBE questionnaire. Scale scores were then calculated using observed and imputed items together. For compliance (i.e. dosage), all missing values were imputed as zero, as medication was dispensed at weekly study visits. Therefore, if a participant had missing data from the study visit, they did not receive the medication dispensation. When considering the outcome variable, 17.75% of participants were missing on the TLFB for all days in the maintenance phase. We took two approaches accounting for this missingness: (i) imputing missing values based on predictor scores and (ii) excluding individuals missing on the outcome^2^. We used a validation analysis to estimate bias for each model, and the models with missing outcomes excluded showed less bias than those with imputed outcomes. As such, the models with individuals who dropped out excluded are reported in the primary manuscript, and the models with imputed outcomes are reported in the Appendix.

## Results

### Percent change in heavy drinking days

**Figure 1:**
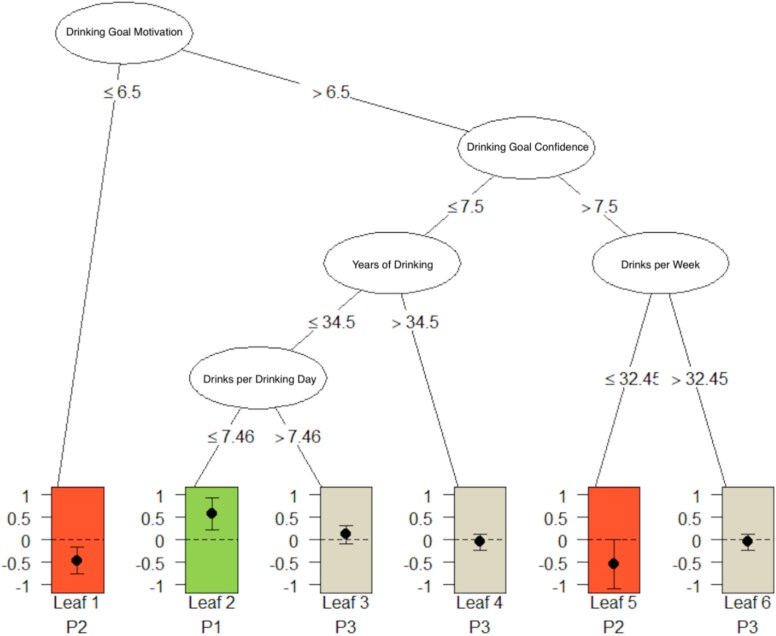
Percent change in heavy drinking days predicted by baseline variables. Treatment responders (indicated in green) were participants with higher motivation to meet their individual drinking goal, lower confidence at achieving their drinking goal, and fewer drinks per drinking day at baseline (Leaf 2). Iatrogenic responders, i.e. participants who responded better to placebo than to GE-XR (indicated in red), were participants with lower motivation to achieve their individual drinking goal (Leaf 1) or higher motivation to achieve their drinking goal, more confidence in achieving their drinking goal, and fewer drinks per week at baseline (Leaf 5). Nonresponders are indicated in tan (Leaves 3, 4, and 6)

The analysis resulted in a pruned tree with six leaves (see Fig. 1 and Table 1), with each leaf representing a subgroup of participants defined as treatment responders, iatrogenic responders, or nonresponders. Treatment responder leaves indicate that treatment with GE-XR was more effective at reducing HDD relative to placebo (displayed in green), whereas iatrogenic responder leaves indicate that treatment with placebo was more effective at reducing HDD relative to GE-XR (displayed in red). Leaves where there were no differences in HDD between treatment groups were defined as nonresponders and are displayed in gray.

**Table 1:** Percent change HDD results (eight-leaf maximum model).

	Placebo			GE-XR			Comparison		
Leaf	*n*	Mean	SD	*n*	Mean	SD	ΔMean	SE	P
1	10	0.84	0.24	11	0.38	0.43	0.46	0.15	2
2	17	0.21	0.62	13	0.80	0.25	−.59	0.18	1
3	29	0.54	0.36	26	0.66	0.40	−0.13	0.10	3
4	37	0.59	0.38	34	0.54	0.40	0.05	0.09	3
5	10	0.89	0.14	10	0.35	0.86	0.53	0.28	2
6	30	0.65	0.35	51	0.60	0.45	0.05	0.10	3

Leaf = leaf number. *n* = number of participants in each condition in each leaf. Mean = observed mean on proportion change in HDD. SD = observed standard deviation on proportion change in HDD. ΔMean = observed mean difference in proportion change in HDD between placebo and GE-XR. SE = estimated standard error of the observed mean difference in proportion change in HDD between GE-XR and placebo. P1 = predicted greater reduction in proportion change in HDD among those in GE-XR condition than placebo. P2 = predicted greater reduction in proportion change in HDD among those in placebo condition than GE-XR. P3 = predicted no difference between GE-XR and placebo conditions in proportion change in HDD.

The tree branches indicate the characteristics of the participant subgroups (leaves). Only one leaf contained treatment responders. The tree was first divided on the participant’s motivation for their individually selected drinking goal (e.g. abstinence, drinking reduction, no goal). Participants with higher motivation for their selected drinking goal but lower confidence in their ability to achieve their goal, fewer years of drinking, and fewer drinks per drinking day at baseline were considered treatment responders to GE-XR (Leaf 2). Participants in this leaf had a greater change in percent heavy drinking days when treated with GE-XR compared to similar participants treated with placebo. In contrast, those who had lower motivation for their individually selected drinking goal (Leaf 1) and those with higher motivation to achieve their goal, higher confidence in achieving their goal, and fewer drinks per week at baseline (Leaf 5) were iatrogenic responders, such that they had a greater change in percent heavy drinking days when treated with placebo compared to similar participants treated with GE-XR. The first nonresponder leaf consisted of individuals with higher motivation to achieve their drinking goal, higher confidence in achieving their goal, and greater drinks per week at baseline (Leaf 6). The other nonresponder leaves consisted of individuals with higher motivation to achieve their drinking goal, lower confidence in achieving their goal, and either a greater number of years of drinking (Leaf 4) or fewer number of years drinking and greater drinks per drinking day at baseline (Leaf 3). These leaves consist of predictors that did not favor treatment with GE-XR or placebo over the other in regards to percent change in heavy drinking days.

### Raw change in heavy drinking days

**Figure 2:**
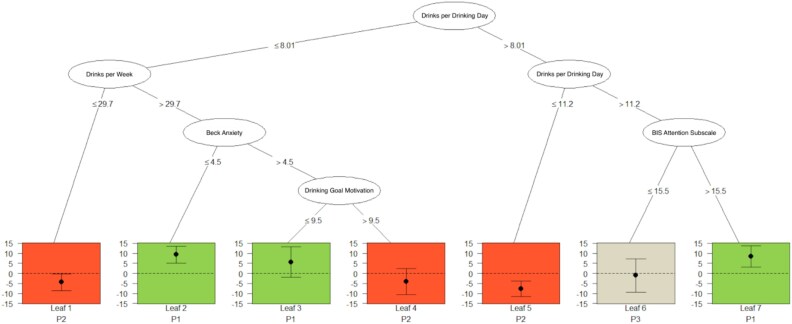
Raw change in heavy drinking days predicted by baseline variables. Treatment responders (indicated in green) were participants with a greater number of drinks per drinking day and more baseline attentional impulsivity (Leaf 7), or fewer number of drinks per drinking day, greater number of drinks per week, and lower baseline anxiety (Leaf 2), or participants with fewer number of drinks per drinking day, greater number of drinks per week, more baseline anxiety, and lower motivation to achieve their individual drinking goal (Leaf 3). Iatrogenic responders, i.e. participants who responded better to placebo than to GE-XR (indicated in red), were participants with fewer drinks per drinking day at baseline and less drinks per week at baseline (Leaf 1); individuals with fewer drinks per drinking day at baseline, greater drinks per week at baseline, greater anxiety as baseline, and more motivation to achieve their drinking goal (Leaf 4); or individuals with an initial greater number of drinks per drinking day at baseline on first split but fewer drinks per drinking day at baseline on second split (Leaf 5). Nonresponders are indicated in tan (Leaf 6).

The raw change model resulted in a pruned tree with seven leaves and identified three treatment responder leaves, three iatrogenic responder leaves, and one nonresponder leaf (Fig. 2 and Table 2). The first two treatment responder leaves consisted of individuals with fewer drinks per drinking day at baseline, but greater drinks per week at baseline, and either lower levels of baseline anxiety (Leaf 2) or higher levels of baseline anxiety and less motivation to achieve their drinking goal (Leaf 3). The other treatment responder leaf consisted of individuals with a greater number of drinks per drinking day at baseline and higher baseline attentional impulsivity, reflecting a greater inability to focus on current tasks (Leaf 7). Participants in these leaves (2, 3, and 7) had a greater raw change in heavy drinking days when treated with GE-XR compared to similar participants treated with placebo. The first iatrogenic leaf consisted of individuals with fewer drinks per drinking day at baseline and fewer drinks per week at baseline (Leaf 1). The second iatrogenic leaf consisted of individuals with fewer drinks per drinking day at baseline but greater drinks per week at baseline, higher levels of baseline anxiety, and greater motivation to achieve their drinking goal (Leaf 4). The final iatrogenic leaf consisted of two splits on baseline drinks per drinking days, with the first indicating a greater number drinks per drinking day (>8.01) and the second indicating fewer drinks per drinking day (<11.2) (Leaf 5). Iatrogenic responders had a better response to treatment with a placebo compared to similar individuals treated with GE-XR. The nonresponder leaf consisted of a greater number of drinks per drinking day at baseline and lower scores on attentional impulsivity at baseline, indicating a greater ability to stay on task (Leaf 6).

**Table 2:** Raw change HDD results (eight-leaf maximum model).

	Placebo			GE-XR			Comparison		
Leaf	*n*	Mean	SD	*n*	Mean	SD	ΔMean	SE	P
1	17	1.47	5.98	12	6.08	5.26	4.39	2.15	2
2	23	8.65	7.82	31	18.03	7.87	−9.38	2.16	1
3	12	6.50	9.51	19	12.11	11.12	−5.61	3.88	1
4	10	14.80	9.39	12	1.83	6.16	3.97	3.33	2
5	42	17.21	8.65	34	9.62	8.37	7.60	1.97	2
6	12	11.83	1.82	16	1.81	11.30	1.02	4.24	3
7	17	8.88	8.34	21	17.33	8.37	−8.45	2.73	1

Leaf = leaf number. n = number of participants in each condition in each leaf. Mean = observed mean on raw change in HDD. SD = observed standard deviation on raw change in HDD. ΔMean = observed mean difference in proportion change in HDD between placebo and GE-XR. SE = estimated standard error of the observed mean difference in raw change in HDD between GE-XR and placebo. P1 = predicted greater reduction in raw change in HDD among those in GE-XR condition than placebo. P2 = predicted greater reduction in raw change in HDD among those in placebo condition than GE-XR. P3 = predicted no difference between GE-XR and placebo conditions in raw change in HDD.

### Validation analysis

The greatest risk of bias in a QUINT analysis is overestimating the difference in effect size between groups given the algorithm focuses on identifying groups with very large positive and negative effect sizes. Dusseldorp and Van Mechelen (2014) propose a validation analysis appropriate for small sample sizes (*N* < 400) to evaluate the likelihood of bias for a specific analysis. Bootstrap samples (*B* = 1000) are taken from the original data, and each is used to fit a QUINT model with the same number of leaves as the optimal number from the original data. In our case, we used seven leaves for raw change in heavy drinking days and six leaves for percent change in heavy drinking days. For each bootstrap sample, the difference between the largest and smallest effect size is calculated and compared to the difference between the largest and smallest effect size when applying the QUINT model fit with the bootstrap sample to the original data. The average of these values ${\overline{O}}_{range}$ indicates the average optimism of the QUINT model, where positive values would indicate that the model is overestimating the range of effect sizes, negative values would indicate underestimation, and values close to zero indicate no bias. We calculated ${\overline{O}}_{range}$= 1.33 for raw change, indicating an overestimation, whereas the largest difference between groups in the model is 16.98, meaning the differences between the largest and smallest effect size are estimated to be inflated by ~61%. We calculated ${\overline{O}}_{range}$ = 0.64 for percent change, whereas the largest difference between groups in the model is 1.12, meaning the difference between the largest and smallest effect sizes is estimated to be inflated by ~57%. These statistics were also used to select between the models that imputed outcome scores or excluded individuals who were missing on the outcome.

### Comparison of raw change and percent change models

**Figure 3:**
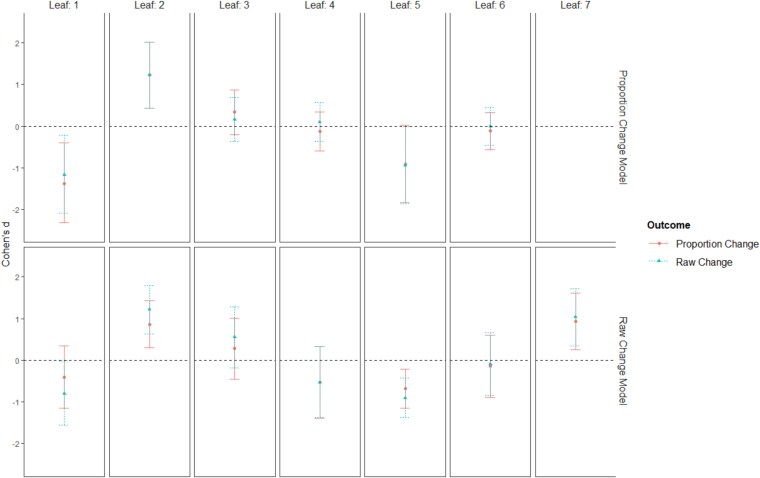
QUINT model comparison. Figure 3 compares the tree identified from each QUINT model against the alternate outcome. Each graph shows the treatment effect with a 95% confidence interval on each outcome (as indicated by the color, type, and shape of the indicator) using the model trained with each outcome variable (rows). The *y*-axis is the treatment effect size (Cohen’s *d*), where positive indicates that gabapentin is predicted to reduce drinking outcomes more than placebo, and negative is the reverse. Each leaf of the model is represented in the columns of the graph. Regardless of the outcome (percent change or raw change), the models provide similar results.

The raw change and percent change models selected different variables and split participants into different leaves, making it difficult to evaluate from the QUINT models directly how much the choice of outcome variables matters to developing strong prediction. However, as in Grodin *et al*. (2024), each tree can be used to subdivide the participants and the observed effect size (Cohen’s *d*) on each outcome can be used to evaluate whether each tree provides similar results across each outcome. Figure 3 provides a visualization of this process where participants are split up based on the leaves from each model (Percent Change 6 leaf and Raw Change 7 leaf). The results show that regardless of the model, the outcome effect sizes are very similar across leaves, meaning that while the two models selected different variables and split participants differently, they do a good job of predicting treatment outcomes on the other outcome variable. The percent change model seems to provide a closer match of the percent change outcome and raw change outcome than the raw change model, but both models provide similar results.

## Discussion

Gabapentin is an accessible and safe medication that is widely used for a range of off-label indications including AUD. Over the past decade, a host of well-powered state-of-art clinical trials of gabapentin for AUD have been conducted. Two of these trials produced positive results (Mason *et al*. 2014; Anton *et al*. 2020) with the Anton *et al*. (2020) trial identifying the high-alcohol withdrawal group as the likely responders. The confirmatory trial of gabapentin for AUD used a prodrug formulation, namely, GE-XR, and this trial resulted in null findings (Falk *et al*. 2019). To reconcile the literature on the clinical efficacy of gabapentin for AUD, this study conducted a machine learning analysis of GE-XR to identify treatment responders, nonresponders, and iatrogenic responders using baseline clinical and demographic data.

Results for the percent change in heavy drinking days outcome identified a subgroup of responders characterized by higher motivation for reaching their drinking goal, lower confidence to achieve their drinking goal, and fewer drinks per drinking day at baseline. These findings suggest that motivation for drinking change combined with a lack of self-efficacy and fewer drinks per drinking day at baseline characterizes a subgroup of responders. Clinically, this may be a group that is well suited for outpatient care on the basis of their drinking levels at baseline and motivation for change yet report lower self-confidence to make change, thus requiring a medication that can assist in making change. Motivation for change has long been identified as a critical factor in the success of treatment for AUD (DiClemente *et al*. 1999), and more recently, pharmacotherapy trials noted differences between treatment seekers and nontreatment seekers as a plausible surrogate indicator of the underlying motivation for change (Ray *et al*. 2017; Lee *et al*. 2019; Goodyear *et al*. 2021). In fact, motivation may be critical to influencing individuals to seek treatment and complete treatment, as well as to make successful long-term changes in their drinking (DiClemente and Scott 1997). In light of the present findings and the growing interest in addressing the placebo effect in AUD trials, considering motivation for change as an inclusion/exclusion variable in clinical trials may be warranted (e.g. include individuals with high motivation for change).

A benefit of the QUINT approach is the identification of iatrogenic responders. In these analyses, a subgroup of iatrogenic responders was characterized by (i) lower levels of motivation to reach drinking goals or (ii) higher motivation, higher confidence in their ability to reach goals, and lower drinks per week at baseline. It possible that the highly motivated group, with high self-efficacy and lower levels of baseline drinking may be prone to a strong placebo response, which could explain the iatrogenic effects of GE-XR in this trial.

Additional analyses considered raw change in heavy drinking days as the outcome. In this case, likely responders to GE-XR were individuals with (i) fewer drinks per day at baseline, greater drinks per week, and lower anxiety or (ii) fewer drinks per day at baseline, greater drinks per week combined with higher anxiety and lower motivation for change, or (iii) greater drinks per drinking day at baseline and higher attentional impulsivity In sum, the raw change outcome analyses emphasize motivation for drinking change, baseline drinking, and anxiety levels as predicting response to GE-XR. This is notable since gabapentin has known anti-anxiety effects in AUD samples (Leung *et al*. 2015).

Iatrogenic responders for the raw change analysis consisted of (i) individuals with fewer drinks per drinking day at baseline and fewer drinks per week at baseline or (ii) participants reporting fewer drinks per drinking day at baseline, greater drinks per week, higher anxiety, and greater motivation for change or (iii) participants reporting roughly between 8 and 11 drinks per drinking day at baseline. It appears that iatrogenic responders using the raw change in heavy drinking day as an outcome were largely identified on the basis of drinking levels at baseline.

Across the two outcomes, our findings suggest that responders had fewer drinks per drinking day or fewer drinking days, higher motivation to reach their drinking goal, lower confidence in their ability to reach drinking goals, lower anxiety, or higher anxiety coupled with lower motivation for change. There was a group of responders in the raw change in heavy drinking day analysis that was characterized by higher cognitive impulsivity and greater drinks per drinking day at baseline. This pattern stands in partial contrast with the findings by Laska *et al*. (2020), in which likely responders had higher heavy drinking days, lower levels of anxiety, depression, and mood disturbance and higher levels of cognitive and motor impulsivity. Our findings are most consistent with Laska *et al*. (2020), insofar as we consider our analyses of raw change in heavy drinking, in which case responders had higher cognitive impulsivity and greater drinks per drinking day at baseline. Consistent with Laska *et al*. (2020), lower levels of anxiety and greater drinks per drinking day were predictive of a favorable response to GE-XR for the outcome of raw change in heavy drinking days. While the raw change in heavy drinking output has resulted in more consistent findings than those of the previous machine learning study in this dataset, our analyses examining percent change in heavy drinking days implicated motivation for change and self-efficacy (i.e. confidence in the ability to reach drinking goal) as predictors of a favorable response to GE-XR.

It is plausible that different approaches to missing data (e.g. Laska *et al*. employed chained equations used for imputation) contributed to the differences observed between Laska *et al*. (2020) and the results present herein. Nevertheless, given the clinical relevance of gabapentin in general, and this confirmatory trial in particular, it is critical to consider these findings in combination. Multiple studies using different machine learning methods are valuable as these novel “analytic tools” become available and are applied to medication development for AUD.

The QUINT models for the two outcomes identified several of the same predictors (e.g. baseline drinking, motivation to reach drinking goal). The models showed consistency when the alternate model was used to predict each outcome. In other words, despite the fact that the models selected slightly different variables as predictors, both models predict similar treatment outcomes with similar effect sizes. This level of consistency is an important feature of the QUINT models and should be considered in future applications to clinical trials.

Pharmacotherapies are unlikely to be globally effective but rather may work for specific individuals, i.e. personalized medicine (Lohoff 2020). A plausible direction for the use of QUINT with RCTs, is to use a QUINT analysis to identify groups of treatment responders, and to use this information to plan future RCTs with specific subpopulations. To that end, the effect size estimates from the QUINT analysis can inform power analyses for more targeted RCTs.

This study should be considered in view of its strengths and limitations. Study strengths include the use of a large, multisite clinical trial, the ability to identify iatrogenic responders in addition to treatment responders, and the ability to simultaneously evaluate moderators in the model. However, the QUINT model has limitations, namely, interpreting high-level interactions (e.g. trees with seven leaves) is difficult and may limit clinical utility for complex models. The QUINT models were sensitive to the outcome selection, in that the two outcomes generated slightly different sets of predictors. However, the model comparisons indicated that the effect sizes were similar when the alternate outcome was used to probe the model. The QUINT method did not use training and testing data, as is often recommended for machine learning models; however, validation analyses were used to evaluate the potential for bias in the range of effect size estimates, as is recommended (Dusseldorp and Van Mechelen 2014). Additional limitations are associated with the *post hoc* nature of this data analysis, such that design decisions in the parent trial influence the results herein. Specifically, the Falk *et al*. (2019) trial required 3 days of abstinence at randomization, which limited the inclusion of individuals unable to maintain abstinence prior to enrollment and lowered the representation of withdrawal symptoms.

In closing, this study identified key factors that are associated with the efficacy (or iatrogenic effects) of GE-XR for AUD. Such factors are baseline drinking levels, motivation for change, confidence in their ability to reach drinking goals (i.e. self-efficacy), cognitive impulsivity, and anxiety levels. Baseline anxiety level and baseline drinking level are predictors associated with the protracted withdrawal syndrome, which has been implicated in the clinical response to gabapentin (Mason *et al*. 2018). Nevertheless, additional predictors were identified in our analyses, namely, motivation for change and self-efficacy. These constructs have a long history in the alcohol research field as determinants of treatment seeking, compliance with treatment, and long-term changes to alcohol use (DiClemente and Scott 1997; DiClemente *et al*. 1999). As such, it is critical to consider these factors in efforts to identify treatment responders, as well as in the design of clinical trials for AUD.

While the resulting patterns identified through machine learning methods are complex, they underscore the realities of clinical practice whereby a confluence of factors predisposes participants to a favorable clinical response to pharmacotherapy. In this study, as in Laska *et al*. (2020), variables associated with protracted withdrawal from alcohol were systematically identified as predictors of clinical response in this confirmatory trial of GE-XR. This results in a nuanced, yet generally consistent, role for baseline drinking levels, motivation, self-efficacy, and anxiety levels to play a role in clinical response to GE-XR for the treatment of AUD.

## Data Availability

The data that support the findings of this study are available from the corresponding author, [LAR], upon reasonable request.

## Footnotes

1 This differs from the original clinical trial that coded these values as one point higher than the original scale. For example, if an item ranged from 0 to 3, “refuse to answer” was coded as a 4.

2 The original clinical trial imputed any missing days on the TLFB as heavy drinking days at both baseline and follow-up (Falk *et al*. 2019)

## References

[ref1a] American Psychiatric Association, DSM-5 Task Force . Diagnostic and statistical manual of mental disorders: DSM-5™ (5th ed.). American Psychiatric Publishing, Inc., 2013. 10.1176/appi.books.9780890425596

[ref1] Anton RF, Myrick H, Baros AM. et al. Efficacy of a combination of flumazenil and gabapentin in the treatment of alcohol dependence: Relationship to alcohol withdrawal symptoms. J Clin Psychopharmacol. 2009;29:334–42. 10.1097/JCP.0b013e3181aba6a4. 19593171

[ref2] Anton RF, Myrick H, Wright TM. et al. Gabapentin combined with naltrexone for the treatment of alcohol dependence. Am J Psychiatry. 2011;168:709–17. 10.1176/appi.ajp.2011.10101436. 21454917 PMC3204582

[ref3] Anton RF, Latham P, Voronin K. et al. Efficacy of gabapentin for the treatment of alcohol use disorder in patients with alcohol withdrawal symptoms: A randomized clinical trial JAMA Intern Med. 2020;180:728–36. 10.1001/jamainternmed.2020.0249. 32150232 PMC7063541

[ref4] Beck AT, Epstein N, Brown G. et al. Beck anxiety inventory J Consult Clin Psychol. 1993;61:194–8.8473571

[ref5] Beck AT, Steer RA, Brown G. Beck depression inventory–II Psychol Assess. 1996.

[ref6] Buysse DJ, Reynolds CF III, Monk TH. et al. The Pittsburgh sleep quality index: A new instrument for psychiatric practice and research Psychiatry Res. 1989;28:193–213. 10.1016/0165-1781(89)90047-4. 2748771

[ref7] Devine EG, Ryan ML, Falk DE. et al. An exploratory evaluation of take control: A novel computer-delivered behavioral platform for placebo-controlled pharmacotherapy trials for alcohol use disorder Contemp Clin Trials. 2016;50:178–85. 10.1016/j.cct.2016.08.006. 27521807 PMC5065069

[ref8] DiClemente CC, Scott CW. Stages of change: Interactions with treatment compliance and involvement NIDA Res Monogr. 1997;165:131–56. 9243549

[ref9] DiClemente CC, Bellino LE, Neavins TM. Motivation for change and alcoholism treatment Alcohol Res Health. 1999;23:86–92. 10890801 PMC6760428

[ref10] Dusseldorp E, Van Mechelen I. Qualitative interaction trees: A tool to identify qualitative treatment–subgroup interactions Stat Med. 2014;33:219–37. 10.1002/sim.5933. 23922224

[ref11] Dusseldorp E, Doove L, Van Mechelen I. Quint: An R package for the identification of subgroups of clients who differ in which treatment alternative is best for them Behav Res Methods. 2016;48:650–63. 10.3758/s13428-015-0594-z. 26092391 PMC4891398

[ref12] Falk DE, Ryan ML, Fertig JB. et al. Gabapentin Enacarbil extended-release for alcohol use disorder: A randomized, double-blind, placebo-controlled, multisite trial assessing efficacy and safety Alcohol Clin Exp Res. 2019;43:158–69. 10.1111/acer.13917. 30403402 PMC6317996

[ref13] Goodyear K, Vasaturo-Kolodner TR, Kenna GA. et al. Alcohol-related changes in behaviors and characteristics from the baseline to the randomization session for treatment and non-treatment seeking participants with alcohol use disorder Am J Drug Alcohol Abuse. 2021;47:760–8. 10.1080/00952990.2021.1961799. 34582281 PMC8711071

[ref14] Grodin EN, Montoya AK, Cruz A. et al. Identifying treatment responders to Varenicline for alcohol use disorder using two machine-learning approaches Clin Psychol Sci. 2024;0:21677026231169922. 10.1177/21677026231169922.

[ref15] Guglielmo R, Martinotti G, Quatrale M. et al. Topiramate in alcohol use disorders: Review and update CNS Drugs. 2015;29:383–95. 10.1007/s40263-015-0244-0. 25899459

[ref16] Hendershot CS, Wardell JD, Samokhvalov AV. et al. Effects of naltrexone on alcohol self-administration and craving: Meta-analysis of human laboratory studies Addict Biol. 2017;22:1515–27. 10.1111/adb.12425. 27411969 PMC6139429

[ref17] Huang LL, Wright JA, Fischer KM. et al. Gabapentinoid prescribing practices at a large Academic Medical Center Mayo Clin Proc Innov Qual Outcomes. 2023;7:58–68. 10.1016/j.mayocpiqo.2022.12.002. 36660177 PMC9842797

[ref18] Kim Y, Hack LM, Ahn ES. et al. Practical outpatient pharmacotherapy for alcohol use disorder Drugs Context. 2018;7:212308–14. 10.7573/dic.212308. 29445407 PMC5804871

[ref19] Laska EM, Siegel CE, Lin Z. et al. Gabapentin Enacarbil extended-release versus placebo: A likely responder reanalysis of a randomized clinical trial Alcohol Clin Exp Res. 2020;44:1875–84. 10.1111/acer.14414. 33460198 PMC7540534

[ref20] Lee MR, Sankar V, Hammer A. et al. Using machine learning to classify individuals with alcohol use disorder based on treatment seeking status EClinicalMedicine. 2019;12:70–8. 10.1016/j.eclinm.2019.05.008. 31388665 PMC6677650

[ref21] Leung JG, Hall-Flavin D, Nelson S. et al. The role of gabapentin in the management of alcohol withdrawal and dependence Ann Pharmacother. 2015;49:897–906. 10.1177/1060028015585849. 25969570

[ref22] Lipkovich I, Dmitrienko A, D'Agostino RB Sr. Tutorial in biostatistics: Data-driven subgroup identification and analysis in clinical trials Stat Med. 2017;36:136–96. 10.1002/sim.7064. 27488683

[ref23] Lohoff FW . Pharmacotherapies and personalized medicine for alcohol use disorder: A review Pharmacogenomics. 2020;21:1117–38. 10.2217/pgs-2020-0079. 32807012 PMC7586357

[ref24] Mariani JJ, Rosenthal RN, Tross S. et al. A randomized, open-label, controlled trial of gabapentin and phenobarbital in the treatment of alcohol withdrawal Am J Addict. 2006;15:76–84. 10.1080/10550490500419110. 16449096

[ref25] Marlatt GA, Curry S, Gordon JR. A longitudinal analysis of unaided smoking cessation J Consult Clin Psychol. 1988;56:715–20. 10.1037/0022-006X.56.5.715. 3192787

[ref26] Martinotti G, Di Nicola M, Tedeschi D. et al. Pregabalin versus naltrexone in alcohol dependence: A randomised, double-blind, comparison trial J Psychopharmacol. 2010;24:1367–74. 10.1177/0269881109102623. 19346279

[ref27] Mason BJ, Quello S, Goodell V. et al. Gabapentin treatment for alcohol dependence: A randomized clinical trial JAMA Intern Med. 2014;174:70–7. 10.1001/jamainternmed.2013.11950. 24190578 PMC3920987

[ref28] Mason BJ, Quello S, Shadan F. Gabapentin for the treatment of alcohol use disorder Expert Opin Investig Drugs. 2018;27:113–24. 10.1080/13543784.2018.1417383. PMC5957503 29241365

[ref29] McNair DM, Lorr M, Droppleman LF . Revised manual for the Profile of Mood States. San Diego, CA: Educational and Industrial Testing Services, 1992.

[ref30] Miller WR . *The Drinker Inventory of Consequences (DrInC): An Instrument for Assessing Adverse Consequences of Alcohol Abuse: Test Manual*. US Department of Health and Human Services, Public Health Service, National, 1995.

[ref31] Myrick H, Malcolm R, Randall PK. et al. A double-blind trial of gabapentin versus lorazepam in the treatment of alcohol withdrawal Alcohol Clin Exp Res. 2009;33:1582–8. 10.1111/j.1530-0277.2009.00986.x. 19485969 PMC2769515

[ref32] Patton JH, Stanford MS, Barratt ES. Factor structure of the Barratt impulsiveness scale J Clin Psychol. 1995;51:768–74. 10.1002/1097-4679(199511)51:6<768::AID-JCLP2270510607>3.0.CO;2-1. 8778124

[ref33] Paulus JK, Kent DM. Predictably unequal: Understanding and addressing concerns that algorithmic clinical prediction may increase health disparities NPJ Digit Med. 2020;3:99. 10.1038/s41746-020-0304-9. 32821854 PMC7393367

[ref34] Ray LA, Bujarski S, Yardley MM. et al. Differences between treatment-seeking and non-treatment-seeking participants in medication studies for alcoholism: Do they matter? Am J Drug Alcohol Abuse. 2017;43:703–10. 10.1080/00952990.2017.1312423. 28426264 PMC6159933

[ref35] Schacht JP, Randall PK, Waid LR. et al. Neurocognitive performance, alcohol withdrawal, and effects of a combination of flumazenil and gabapentin in alcohol dependence Alcohol Clin Exp Res. 2011;35:2030–8. 10.1111/j.1530-0277.2011.01554.x. 21631542 PMC3166540

[ref36] Singleton E, Henningfield J, Tiffany S. *Alcohol Craving Questionnaire: ACQ-Now: Background and Administration Manual*. Baltimore: NIDA Addiction Research Centre, 1994.

[ref37] Sobell, Linda C, Mark B Sobell . Timeline follow-back: A technique for assessing self-reported alcohol consumption. In Measuring Alcohol Consumption: Psychosocial and Biochemical Methods, pp. 41–72. Totowa, NJ: Humana Press, 1992.

[ref38] Sullivan JT, Sykora K, Schneiderman J. et al. Assessment of alcohol withdrawal: The revised clinical institute withdrawal assessment for alcohol scale (CIWA-Ar) Br J Addict. 1989;84:1353–7. 10.1111/j.1360-0443.1989.tb00737.x. 2597811

[ref39] Werner M, Rentz A, Frank L. et al. Participant consequence measures. In: *Paper Presented at the Annual Meeting of the Research Society on Alcoholism*, Vol. 2008. Washington, DC, 2008.

